# Supplementary material for: Alveolar epithelial and vascular CXCR2 mediates transcytosis of CXCL1 in inflamed lungs
Source: Nat Commun. 2025 May 24;16:4846. doi: 10.1038/s41467-025-60174-w (PMC12103508; doi:10.1038/s41467-025-60174-w)
Supplement: Supplementary file 1 — Supplementary Information [file 41467_2025_60174_MOESM1_ESM.pdf]

Alveolar epithelial and vascular CXCR2 mediates transcytosis of CXCL1 in inflamed lungs

Katharina Thomas[1#], Jan Rossaint[1#], Nadine Ludwig[1], Sina Mersmann[1], Niklas Kötting[1], Julia Grenzheuser[1], Lena Schemmelmann[1], Marina Oguama[1], Andreas Margraf[1], Helena Block[1], Katharina Henke[1], Katharina Hellenthal[1], Valbona Mirakaj[2], Volker Gerke[3], Uwe Hansen[4], Karin Gäher[4], Miguel Engelhardt[6], Johannes Roth[5], Johannes Eble[6], Elin Hub[7], Antal Rot[7], Ronen Alon[8], and Alexander Zarbock[1]*

[1] Department of Anesthesiology, Intensive Care and Pain Medicine, University Hospital Münster, 48149 Münster, Germany
[2] Department of Anaesthesiology and Intensive Care Medicine, University Tübingen, 72076, Tübingen, Germany
[3] Institute of Medical Biochemistry, Centre for Molecular Biology of Inflammation (ZMBE), University of Münster, 48149 Münster, Germany.
[4] Institute of Experimental Musculoskeletal Medicine, University Hospital Münster, 48149 Münster, Germany
[5] Institute of Immunology, University Hospital Münster, 48149 Münster, Germany
[6] Institute of Physiological Chemistry and Pathobiochemistry, University of Münster, 48149 Münster, Germany
[7] Centre for Microvascular Research, William Harvey Research Institute, Barts and The London School of Medicine and Dentistry, Queen Mary University of London, London EC1M 6BQ, UK
[8] Department of Immunology and Regenerative Biology, Weizmann Institute of Science, Rehovot, Israel.

# Contributed equally

* Corresponding author

Corresponding author

Alexander Zarbock

Department of Anesthesiology, Intensive Care and Pain Medicine

University Hospital Münster

Albert-Schweitzer-Campus 1, Building A1

48149 Münster, Germany

Phone: +49 (251) 8347252

Fax: +49 (251) 88704

E-mail: zarbock@uni-muenster.de

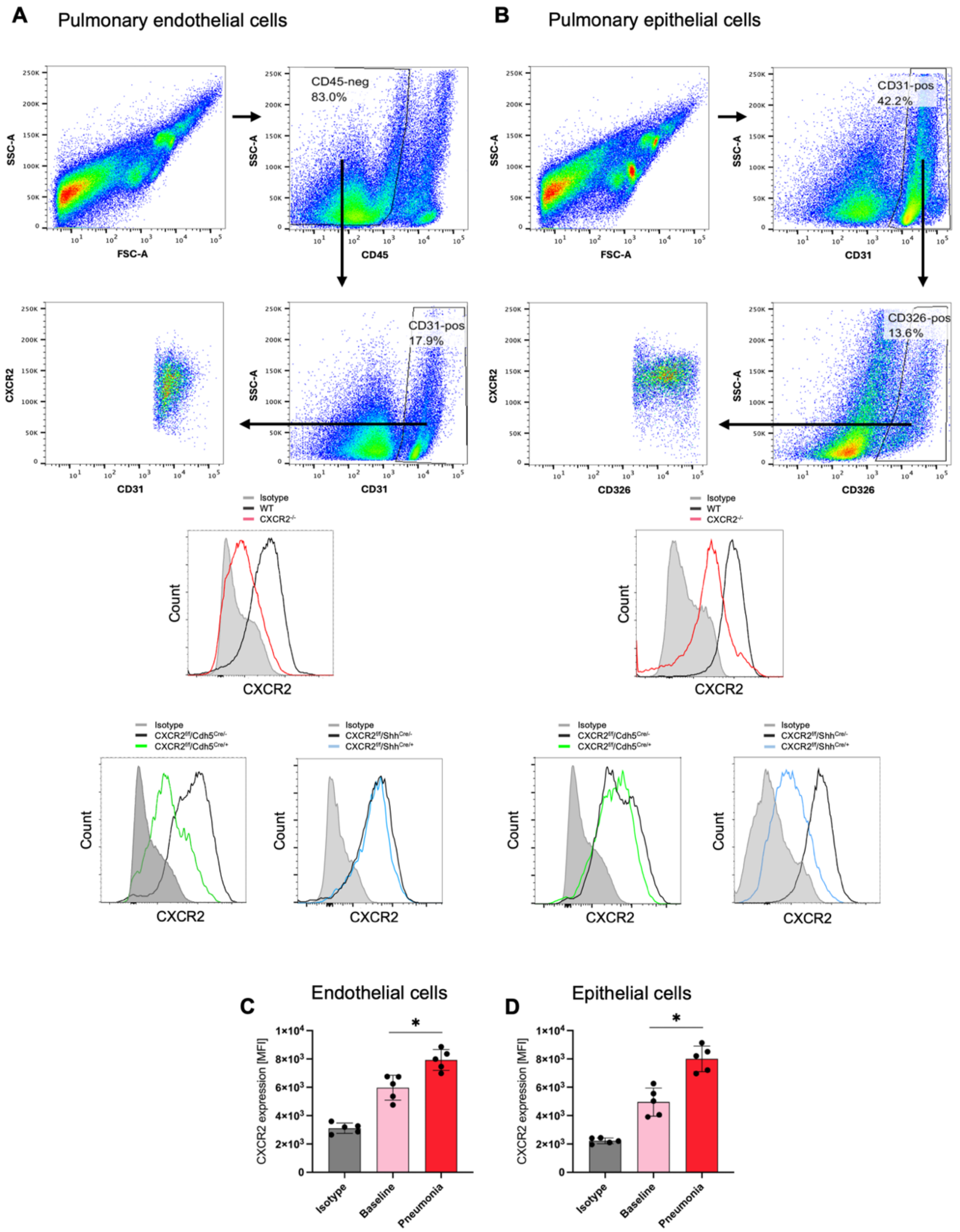

Supplementary Figure 1: CXCR2 is expressed on pulmonary endothelial and epithelial cells. CXCR2 KO mice were intratracheally injected with IL-1β and CXCR2 surface expression was analyzed on alveolar endothelial **(A)** and epithelial **(B)** cells 12 hours later via flow cytometry. Red=CXCR2$^{-/-}$, green=CXCR2$^{fl/fl}$Cdh5^{Cre+}, blue=CXCR2$^{fl/fl}$Shh^{Cre}, WT/Cre^{-} controls=black. Isotype controls are marked in grey. Bacterial pneumonia was induced in WT mice by intratracheal instillation of viable *K. pneumoniae* and CXCR2 expression **(C)** on lung endothelial and **(D)** epithelial cells was analyzed by flow cytometry (n=5 biologically independent mice pre group, age 8-16 weeks, random gender distribution). Data are mean ± SD, one-way ANOVA followed by Bonferroni correction, *p<0.05.

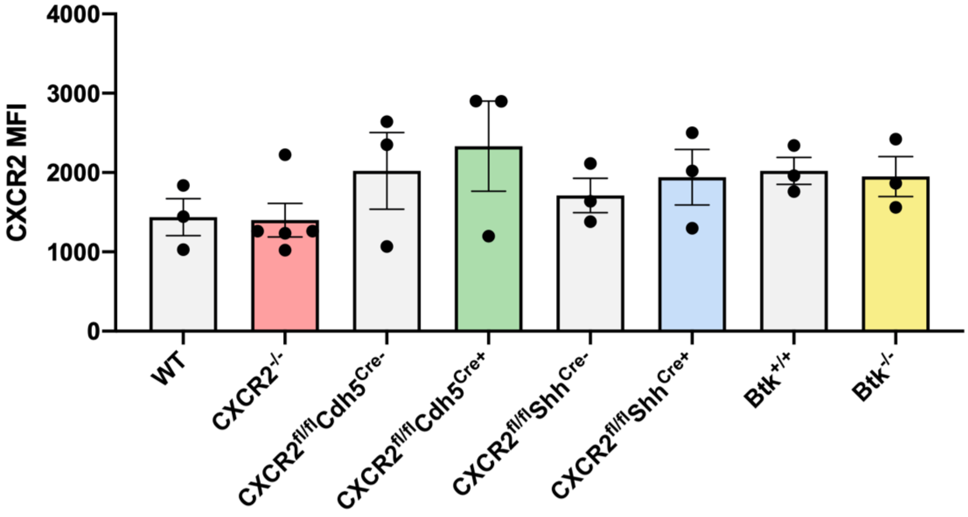

Supplementary Figure 2: CXCR2 expression on circulating neutrophils. Whole blood samples were withdrawn from the indicated mouse lines under baseline conditions and stained for CD45, Ly6G and CXCR2. The mean fluorescence intensity (MFI) of CXCR2 in $CD45^+Ly6G^+$ cells was analyzed by flow cytometry. Data are mean ± SD, n=3-5 biologically independent mice pre group, age 8-16 weeks, equal gender distribution, one-way ANOVA, *p<0.05.

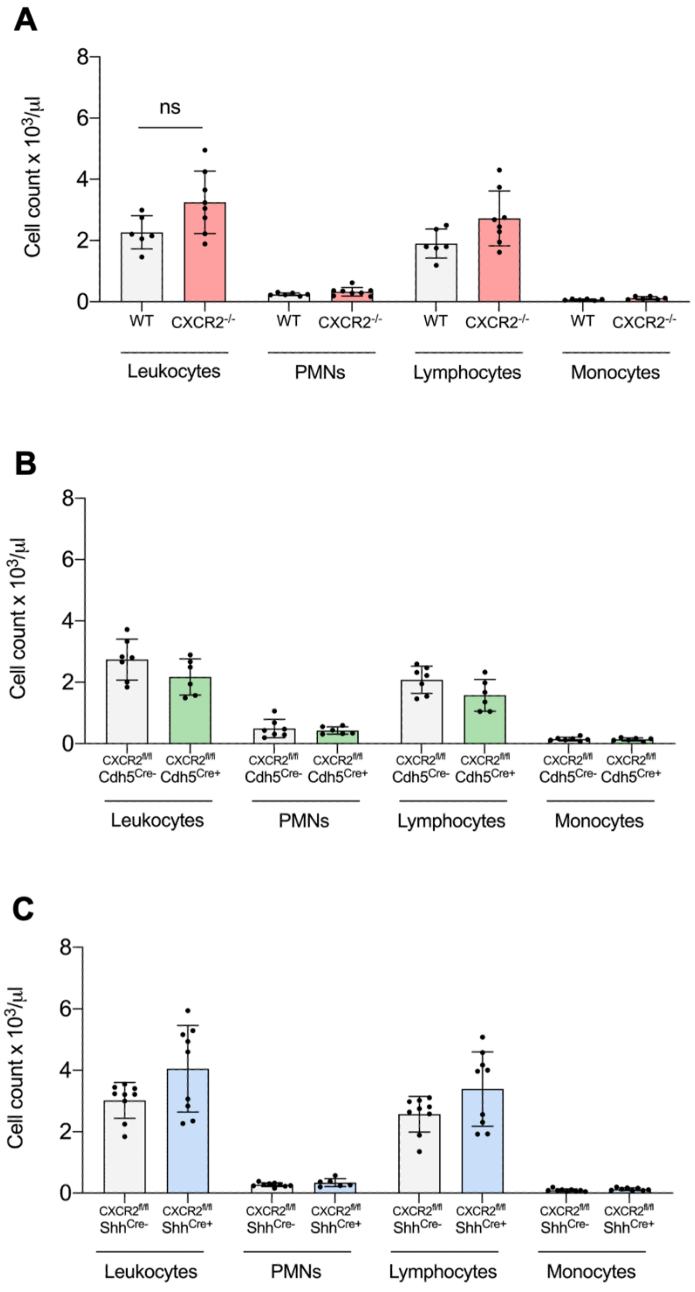

Supplementary Figure 3: Baseline leukocyte counts. Blood was drawn from CXCR2 KO mice and analyzed in a Sysmex haemocytometer. Total leukocyte counts as well as neutrophil, lymphocyte and monocyte counts are displayed for $CXCR2^{-/-}$ mice **(A)**, $CXCR2^{fl/fl}Cdh5^{Cre+}$ mice **(B)**, $CXCR2^{fl/fl}Shh^{Cre+}$ mice **(C)** and respective control mice. Data are mean ± SD, n=6-9 biologically independent mice pre group, age 8-16 weeks, equal gender distribution, one-way ANOVA followed by Bonferroni correction.

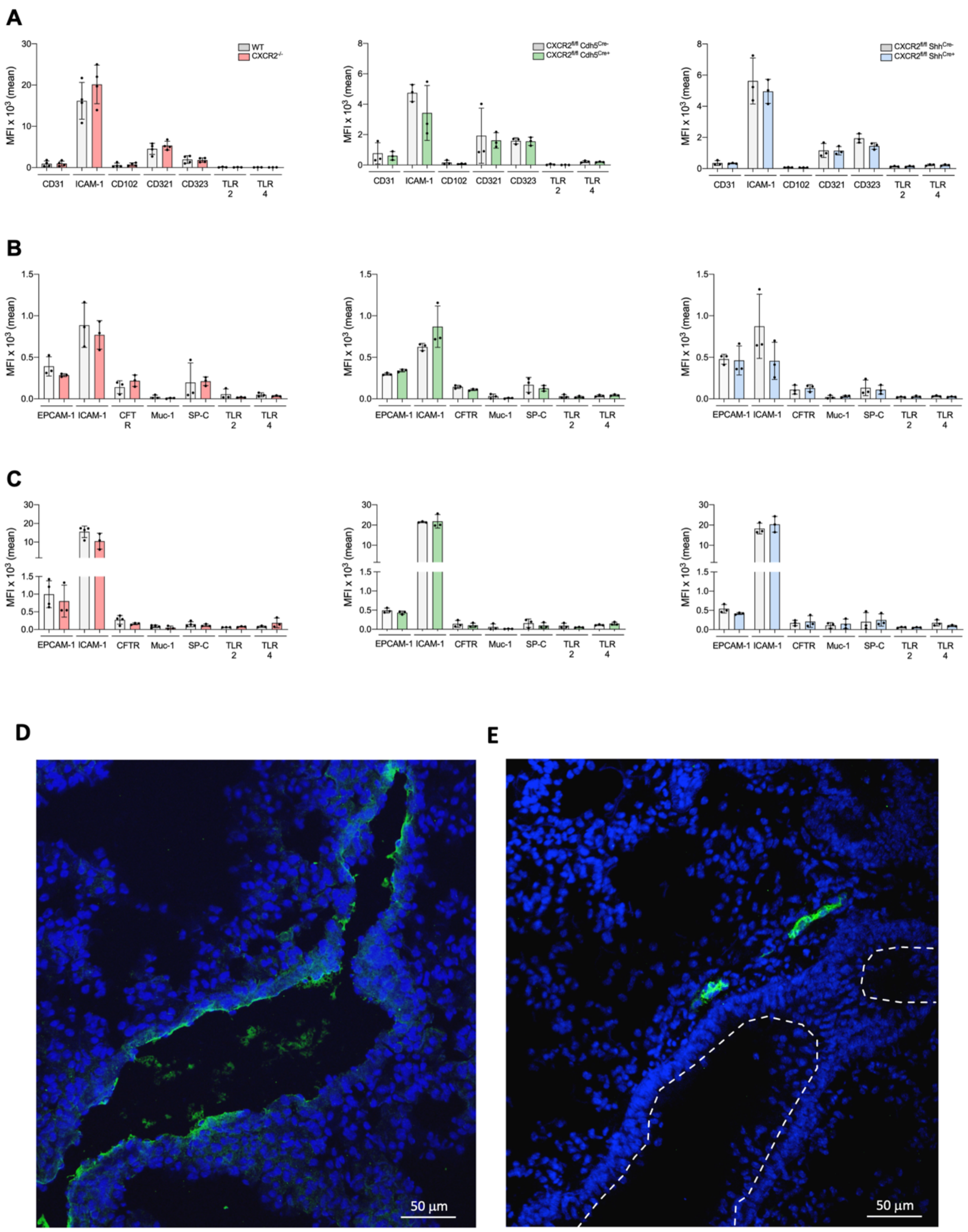

Supplementary Figure 4: Surface marker expression of endothelial and epithelial cells. MLMVECs and AECs were isolated and analyzed for common surface markers by flow cytometry. **(A)** Expression of CD31, CD54, CD102, CD321, CD323, TLR2 and TLR4 was determined on MLMVECs which were isolated from different CXCR2 KO mice (red = CXCR2$^{-/-}$, green = CXCR2$^{fl/fl}$Cdh5^{Cre+}, blue = CXCR2$^{fl/fl}$Shh^{Cre+}, grey = WT/Cre^{-} controls). CD326, CD54, Connexin-43, CFTR, Mucin-1, SP-C, TLR2 and TLR4 expression was analyzed on **(B)** AECs type II (2 days after isolation) and **(C)** type I (7 days after isolation). **(D)** Specific anti-ACKR1 antibody staining associated with the endothelial cell lining of a large vein and erythrocytes in the vessel lumen (green). **(E)** ACKR1 staining (green) in venules lining the bronchial tree in the submucosa of a large bronchus (bronchial lumen outlined by dotted line). Cell nuclei stained with DAPI (blue), scale bars equal 50µm. Data are mean ± SD, n=3-4 biologically independent experiments per group, one-way ANOVA.

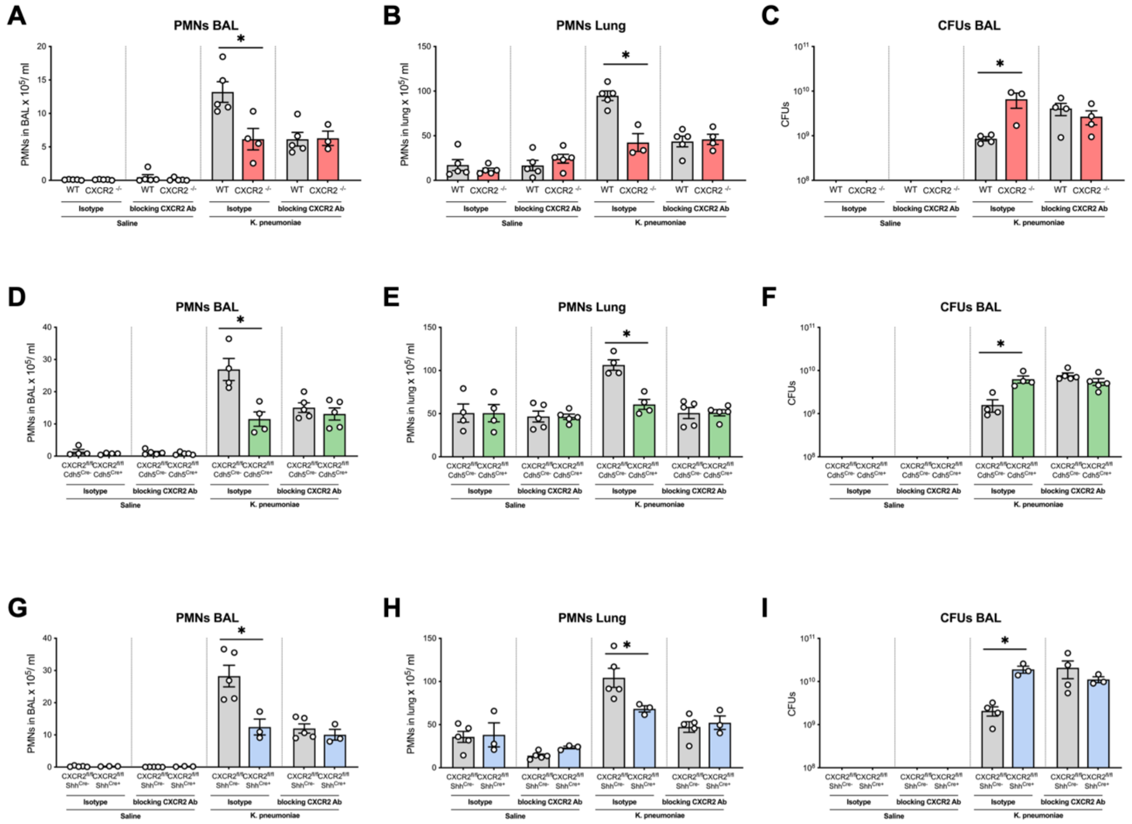

Supplementary Figure 5: Blocking CXCR2 antibody. CXCR2 KO mice (red = $CXCR2^{-/-}$, green = $CXCR2^{fl/fl}Cdh5^{Cre+}$, blue = $CXCR2^{fl/fl}Shh^{Cre+}$) and appropriate WT/Cre^{-} control (grey) mice were intratracheally injected with a blocking CXCR2 antibody (30 µg, R&D) or isotype control and afterwards intratracheally administered with sterile saline or with viable *K. pneumoniae*. Neutrophil recruitment into the BAL **(A, D, G)** and into the lung **(B, E, H)** as well as CFU counts in the BAL **(C, F, I)** were assessed 24 h after infection. Data are mean ± SD, n=3-5 biologically independent mice pre group, age 8-16 weeks, equal gender distribution, one-way ANOVA followed by Bonferroni correction, *p<0.05.

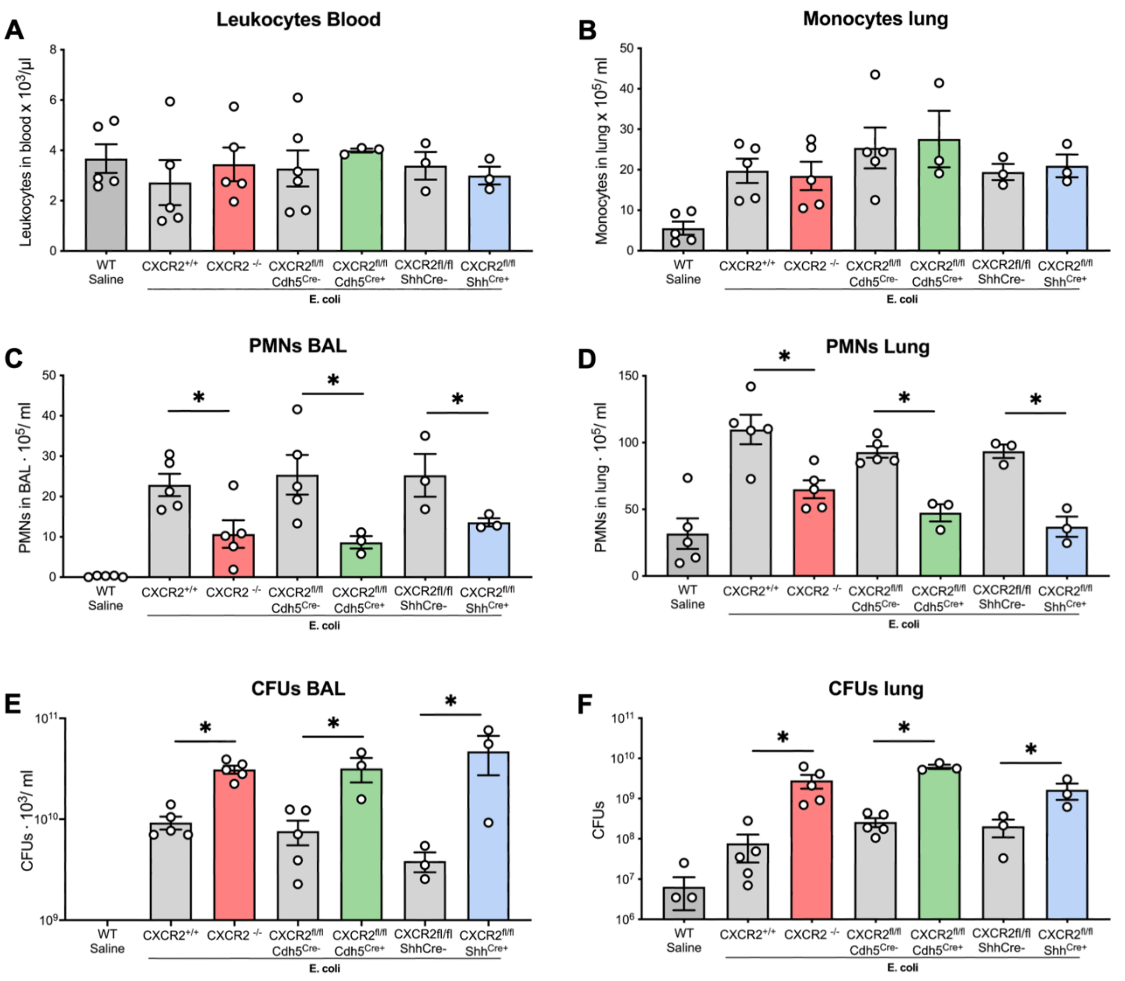

Supplementary Figure 6: *E. coli* pulmonary infection model. CXCR2 KO mice (red = CXCR2$^{-/-}$, green = CXCR2$^{fl/fl}$Cdh5^{Cre+}, blue = CXCR2$^{fl/fl}$Shh^{Cre+}) and appropriate WT/Cre^{-} control (grey) mice were intratracheally administered with sterile saline or with viable *E. coli*. Leukocyte blood counts **(A)** and monocyte counts in the lung **(B)** were analyzed after 24 hours. Neutrophil recruitment into the BAL **(C)** and into the lung **(D)** as well as CFU counts in the BAL **(E)** and the lung tissue **(F)** were assessed 24 hours after infection. Data are mean ± SD, n=3-5 biologically independent mice pre group, age 8-16 weeks, equal gender distribution, one-way ANOVA followed by Bonferroni correction, *p<0.05.

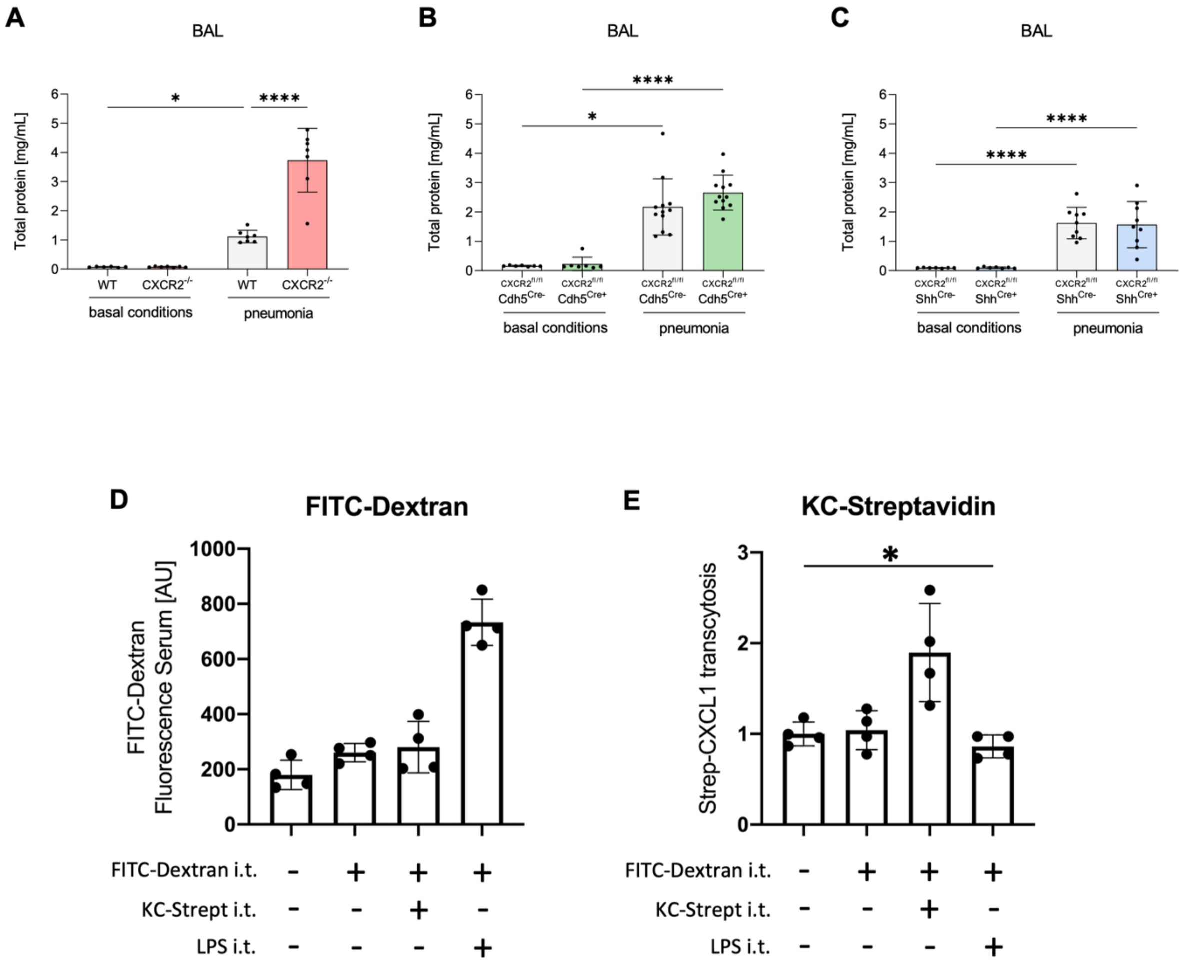

Supplementary Figure 7: Total protein content is elevated in full but not conditional KO mice after lung infection. Total protein concentrations were measured in BAL using a BCA Assay kit under baseline conditions and after *K. pneumoniae*-induced lung infection (pneumonia). **(A)** CXCR2$^{-/-}$(red), **(B)** CXCR2$^{fl/fl}$Cdh5^{Cre+} (green) and **(C)** CXCR2$^{fl/fl}$Shh^{Cre+} (blue) mice were compared with appropriate WT/Cre^{-} control animals. Data are mean ± SD, n=7-12 biologically independent mice pre group, age 8-16 weeks, equal gender distribution. Wildtype mice were intratracheally injected with CXCL1-Streptavidin (MW 8kDa), FITC-Dextran (MW 10kDa) or LPS as a positive control. **(D)** After 4 h, blood samples were obtained and the serum levels of FITC-Dextran were analyzed by photometry. **(E)** The levels of transcytosed streptavidin-coupled CXCL1 were analyzed by an ELISA using a CXCL1 capture antibody and a biotin-HRP conjugated detection antibody. n=4 biologically independent mice pre group, age 8-16 weeks, equal gender distribution, one-way ANOVA followed by Bonferroni correction, *p<0.05, **** p<0.0001.

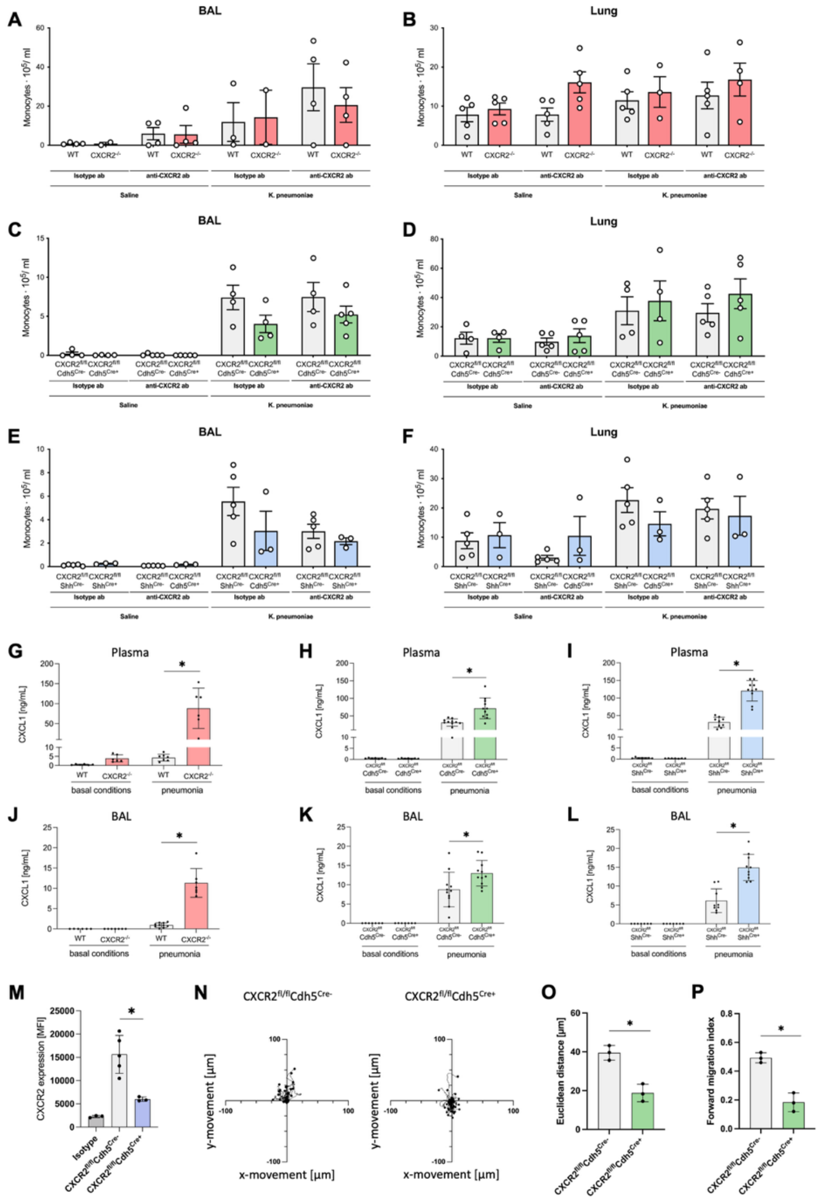

Supplementary Figure 8: Inflammatory monocyte trafficking into the lung during bacterial pneumonia. CXCR2 KO mice (red = $CXCR2^{-/-}$, green = $CXCR2^{fl/fl}Cdh5^{Cre+}$, blue = $CXCR2^{fl/fl}Shh^{Cre+}$) and appropriate WT/Cre^{-} control (grey) mice were injected with a blocking CXCR2 antibody (30 µg, R&D) or isotype control and afterwards intratracheally administered with sterile saline or with viable *K. pneumoniae*. After 24 hours, the numbers of monocytes in the BAL **(A, C, E)** and lung tissue **(B, D, F)** were analyzed by flow cytometry as $CD45^{+}CD11b^{+}CX3CR1^{+}CD115^{+}$ cells (n=3-5 biologically independent mice pre group, age 8-16 weeks, equal gender distribution). CXCR2 KO mice (red = $CXCR2^{-/-}$, green = $CXCR2^{fl/fl}Cdh5^{Cre+}$, blue = $CXCR2^{fl/fl}Shh^{Cre+}$) and appropriate WT/Cre^{-} control (grey) mice were intratracheally administered with sterile saline or with viable *K. pneumoniae*. CXCL1 concentrations in the plasma **(G-I)** and the BAL **(J-L)** were assessed 24 h after infection (n=6-11 biologically independent mice pre group, age 8-16 weeks, equal gender distribution). **(M)** CXCR2 expression levels on neutrophils was analyzed by flow cytometry and **(N-P)** migration of neutrophils was investigated in vitro (n=3 biologically independent samples per group). Data are mean ± SD, one-way ANOVA followed by Bonferroni correction or t-text, *p<0.05.

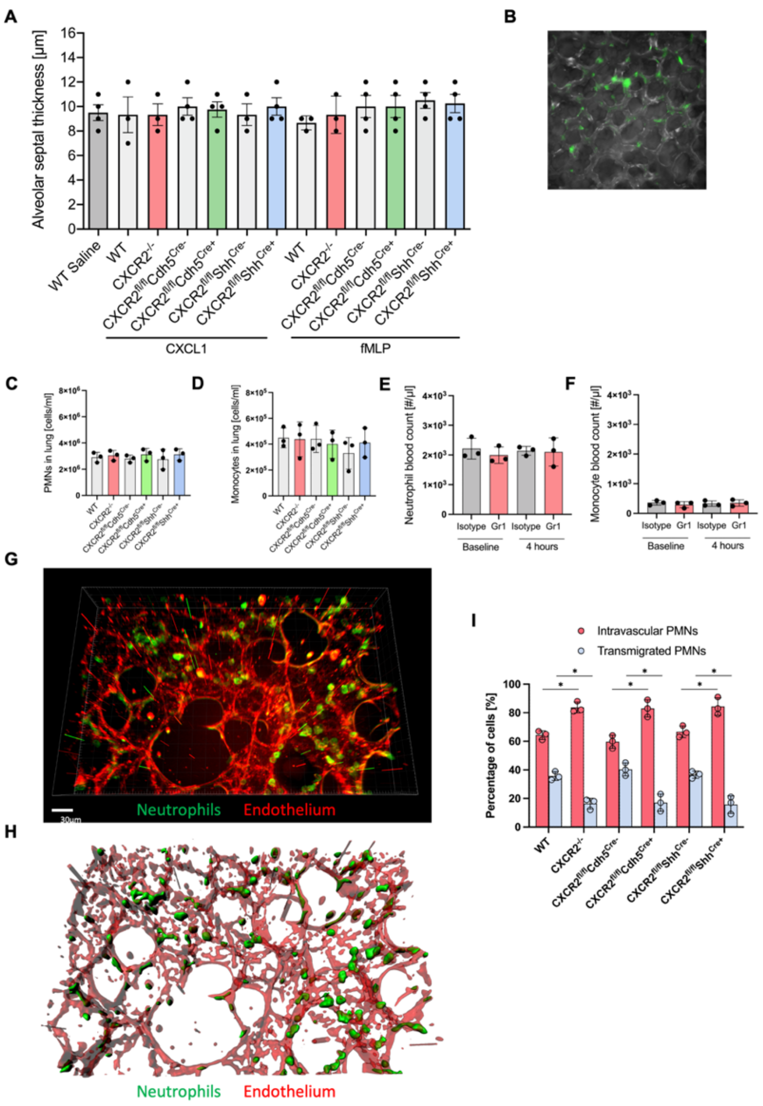

Supplementary Figure 9: Lung septal thickness in lung intravital microscopy. (A) Intravital microscopy of the lung was performed in CXCR2 KO mice (red = CXCR2$^{-/-}$, green = CXCR2$^{fl/fl}$Cdh5^{Cre+}, blue = CXCR2$^{fl/fl}$Shh^{Cre+}) and appropriate WT/Cre^{-} controls (grey) 4 hours after intratracheal injection of sterile saline, CXCL1 or fMLP and the thickness of the alveolar septa (air-to-air distance) was analyzed. **(B)** Lung intravital microscopy of WT mice with Gr1-Alexa488 and CD115-Alexa 568 antibody (exemplary image from n=3 independent experiments). WT mice were intratracheally injected with CXCL1 and administered the Gr1 antibody or isotype control and analyzed the abundancy of **(C)** neutrophils and **(D)** monocytes in the lung by flow cytometry after 4 hours (n=3 biologically independent mice pre group, age 8-16 weeks, random gender distribution). WT mice were administered with 4µg of labeled Gr1 antibody and **(E)** neutrophil and **(F)** monocyte blood counts were analyzed after 4 hours (n=3 biologically independent mice pre group, age 8-16 weeks, random gender distribution). Precision cut lung slices were obtained from CXCR2$^{-/-}$, CXCR2$^{fl/fl}$Cdh5^{Cre+} or CXCR2$^{fl/fl}$Shh^{Cre+} and appropriate WT/Cre^{-} controls 4 hours after intratracheal injection of CXCL1. Exemplary images of **(G)** raw z-stack and **(H)** 3D reconstruction by volume rendering. **(I)** The distribution of neutrophils in the intravascular space and neutrophils emigrated into the extravascular lung tissue was analyzed. Data are mean ± SD, scale bars equal 30 µm, n=3 biologically independent mice pre group, age 8-16 weeks, equal gender distribution, one-way ANOVA followed by Bonferroni correction, *$p<0.05$.

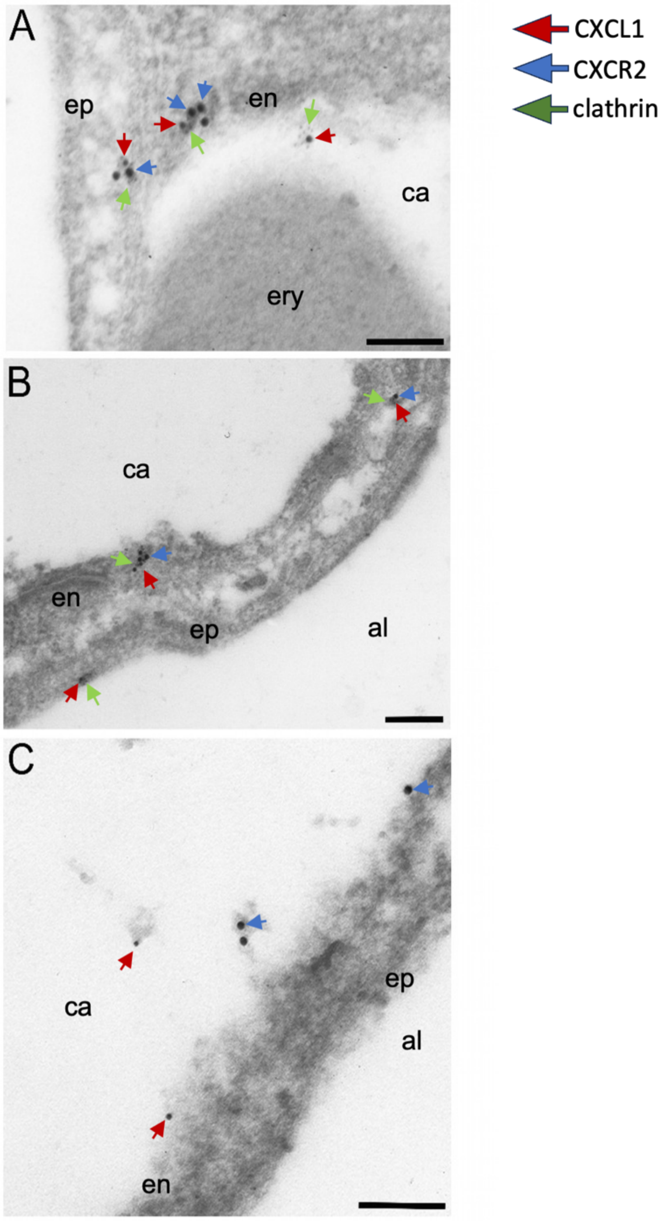

Supplementary Figure 10: CXCL1 and CXCR2 co-localize in pulmonary epithelial and endothelial cells. Colocalization of CXCR2 (blue), CXCL1 (red) and clathrin (green) was analyzed in ultrathin cross-sectioned lung tissue from *K. pneumoniae* infected WT mice via transmission electron microscopy (TEM). **(A)** CXCL1/CXCR2/clathrin complexes at the apical plasma membrane of endothelial cells. **(B)** CXCL1/CXCR2/clathrin complexes in epithelial and endothelial cells. **(C)** CXCL1 and CXCR2 on endothelial protrusions. Abbreviations: ery=erythrocyte, ca=capillary, en=endothelium, ep=epithelium, al=alveolar space. Scale bar: 500 nm
