# Supplementary material for: Alveolar epithelial and vascular CXCR2 mediates transcytosis of CXCL1 in inflamed lungs
Source: Nat Commun. 2025 May 24;16:4846. doi: 10.1038/s41467-025-60174-w (PMC12103508; doi:10.1038/s41467-025-60174-w)
Supplement: Supplementary file 2 — Reporting Summary [file 41467_2025_60174_MOESM2_ESM.pdf]

nature portfolio

Corresponding author(s): Alexander Zarbock

Last updated by author(s): Apr 28, 2025

Reporting Summary

Nature Portfolio wishes to improve the reproducibility of the work that we publish. This form provides structure for consistency and transparency in reporting. For further information on Nature Portfolio policies, see our Editorial Policies and the Editorial Policy Checklist.

Statistics

For all statistical analyses, confirm that the following items are present in the figure legend, table legend, main text, or Methods section.

n/a	Confirmed	
☐	☒	The exact sample size (*n*) for each experimental group/condition, given as a discrete number and unit of measurement
☐	☒	A statement on whether measurements were taken from distinct samples or whether the same sample was measured repeatedly
☐	☒	The statistical test(s) used AND whether they are one- or two-sided<br>*Only common tests should be described solely by name; describe more complex techniques in the Methods section.*
☐	☒	A description of all covariates tested
☐	☒	A description of any assumptions or corrections, such as tests of normality and adjustment for multiple comparisons
☐	☒	A full description of the statistical parameters including central tendency (e.g. means) or other basic estimates (e.g. regression coefficient) AND variation (e.g. standard deviation) or associated estimates of uncertainty (e.g. confidence intervals)
☒	☐	For null hypothesis testing, the test statistic (e.g. *F*, *t*, *r*) with confidence intervals, effect sizes, degrees of freedom and *P* value noted<br>*Give P values as exact values whenever suitable.*
☒	☐	For Bayesian analysis, information on the choice of priors and Markov chain Monte Carlo settings
☒	☐	For hierarchical and complex designs, identification of the appropriate level for tests and full reporting of outcomes
☒	☐	Estimates of effect sizes (e.g. Cohen's *d*, Pearson's *r*), indicating how they were calculated

Our web collection on statistics for biologists contains articles on many of the points above.

Software and code

Policy information about availability of computer code

Data collection	Other than standard software for image aquisition (Zeiss ZEN, 3i SlideBook) and data collection (Microsoft Excel), no specific software or code was used
Data analysis	All statistical calculations were performed using the GraphPad PRISM software (version 9).

For manuscripts utilizing custom algorithms or software that are central to the research but not yet described in published literature, software must be made available to editors and reviewers. We strongly encourage code deposition in a community repository (e.g. GitHub). See the Nature Portfolio guidelines for submitting code & software for further information.

Data

Policy information about availability of data

All manuscripts must include a data availability statement. This statement should provide the following information, where applicable:

- Accession codes, unique identifiers, or web links for publicly available datasets
- A description of any restrictions on data availability
- For clinical datasets or third party data, please ensure that the statement adheres to our policy

All data are included in the Supplementary Information or available from the authors, as are unique reagents used in this Article. The raw numbers for charts and graphs are available in the Source Data file whenever possible.

Research involving human participants, their data, or biological material

Policy information about studies with human participants or human data. See also policy information about sex, gender (identity/presentation), and sexual orientation and race, ethnicity and racism.

Reporting on sex and gender	This study does not incluce research involving human participants, their data, or biological material.
Reporting on race, ethnicity, or other socially relevant groupings	This study does not incluce research involving human participants, their data, or biological material.
Population characteristics	This study does not incluce research involving human participants, their data, or biological material.
Recruitment	This study does not incluce research involving human participants, their data, or biological material.
Ethics oversight	This study does not incluce research involving human participants, their data, or biological material.

Note that full information on the approval of the study protocol must also be provided in the manuscript.

Field-specific reporting

Please select the one below that is the best fit for your research. If you are not sure, read the appropriate sections before making your selection.

[x] Life sciences   [ ] Behavioural & social sciences   [ ] Ecological, evolutionary & environmental sciences

For a reference copy of the document with all sections, see nature.com/documents/nr-reporting-summary-flat.pdf

Life sciences study design

All studies must disclose on these points even when the disclosure is negative.

Sample size	No statistical methods were used to predetermine the sample size. Experiments were performed in biological triplicated n=3 unless otherwise noted. Animal experiments were performed in biological repetitions of at least n=3 unless otherwise noted. In previous studies using related experiments we determined this sample sizes to be sufficient to ensure reproducibility.
Data exclusions	Due to technical issues data were excluded in mice experiments, when the instillation bacteria did not induce any lunf injury or the neutrophil recruitment into the lungs was completely out of scale. Mice then probably had pre-existing conditions.
Replication	All attempts at replication were successful, and standard deviations were within expected ranges.
Randomization	All experimental mice were randomly allocated.
Blinding	In animal experiments blinding was not applicable, as samples were processed identically through standard (e.g. ELISAs) and in some cases automated procedures (e.g. Flow cytometry) that should not bias outcomes.

Reporting for specific materials, systems and methods

We require information from authors about some types of materials, experimental systems and methods used in many studies. Here, indicate whether each material, system or method listed is relevant to your study. If you are not sure if a list item applies to your research, read the appropriate section before selecting a response.

Materials & experimental systems

n/a	Involved in the study	
[ ]	[x]	Antibodies
[x]	[ ]	Eukaryotic cell lines
[x]	[ ]	Palaeontology and archaeology
[ ]	[x]	Animals and other organisms
[x]	[ ]	Clinical data
[x]	[ ]	Dual use research of concern
[x]	[ ]	Plants

Methods

n/a	Involved in the study	
[x]	[ ]	ChIP-seq
[ ]	[x]	Flow cytometry
[x]	[ ]	MRI-based neuroimaging

Antibodies

Antibodies used	CD45-PerCP/Cy5.5 (clone 30/F11, BioLegend), Ly-6B.2-FITC (clone 7/4, Bio-Rad), Gr-1-AF633 (clone RB6-8C5, purified from hybridoma supernatant), AF488-coupled anti-Gr-1 antibody (clone RB6-8C5, purified from hybridoma supernatant), CXCR2 (clone SA044G4, BioLegend), CD31 (clone 390 RUO, BD), EpCAM (clone G8.8, BioLegend), CD31 (clone MEC13.3, BioLegend), CD54 (clone

	YN1/1.7.4, BioLegend), CD102 (clone 3C4, BioLegend), CD321 (clone H202-106, BD Bioscience), CD323 (clone 206928, R&D), TLR2 (clone CB225, BioLegend) and TLR4 (clone MTS510, Invitrogen), CD326 (clone G8.8, BioLegend), CFTR (clone CF3, abcam), Muc-1 (polyclonal, abcam), SP-C (polyclonal, abcam), pan-AKT (clone C67E7, CST), phospho-Akt (clone 193H12, CST), p38 MAPK (#9212, CST), phospho-p38 MAPK (clone 3D7, CST), Clathrin Heavy Chain (clone D3C6, CST), Dynamin (#2342, CST), Caveolin-1 (#3238, CST)
Validation	CD45-PerCP/Cy5.5 (clone 30/F11, BioLegend), Ly-6B.2-FITC (clone 7/4, Bio-Rad), Gr-1-AF633 (clone RB6-8C5, purified from hybridoma supernatant), AF488-coupled anti-Gr-1 antibody (clone RB6-8C5, purified from hybridoma supernatant), CXCR2 (clone SA044G4, BioLegend), CD31 (clone 390 RUO, BD), EpCAM (clone G8.8, BioLegend), CD31 (clone MEC13.3, BioLegend), CD54 (clone YN1/1.7.4, BioLegend), CD102 (clone 3C4, BioLegend), CD321 (clone H202-106, BD Bioscience), CD323 (clone 206928, R&D), TLR2 (clone CB225, BioLegend) and TLR4 (clone MTS510, Invitrogen), CD326 (clone G8.8, BioLegend), CFTR (clone CF3, abcam), Muc-1 (polyclonal, abcam), SP-C (polyclonal, abcam), pan-AKT (clone C67E7, CST), phospho-Akt (clone 193H12, CST), p38 MAPK (#9212, CST), phospho-p38 MAPK (clone 3D7, CST), Clathrin Heavy Chain (clone D3C6, CST), Dynamin (#2342, CST), Caveolin-1 (#3238, CST)

Animals and other research organisms

Policy information about studies involving animals; ARRIVE guidelines recommended for reporting animal research, and Sex and Gender in Research

Laboratory animals	We used 8–12-week-old male C57BL/6 mice which were kept under specific pathogen-free (SPF) conditions.
Wild animals	N/A
Reporting on sex	All experimental mice were randomly allocated for equal gender distribution.
Field-collected samples	N/A
Ethics oversight	All mouse experiments were approved by the North Rhine-Westphalia Office for Nature, Environment and Consumer Protection (“Landesamt für Natur-, Umwelt- und Verbraucherschutz NRW”; reference number 81-02.04.2019.A445). Specific termination criteria were specified in the animal protocol (abnormal pain, abnormal behaviour, high morbidity) and adhered to throughout the experiments.

Note that full information on the approval of the study protocol must also be provided in the manuscript.

Plants

Seed stocks	N/A
Novel plant genotypes	N/A
Authentication	N/A

Flow Cytometry

Plots

Confirm that:

- [x] The axis labels state the marker and fluorochrome used (e.g. CD4-FITC).
- [x] The axis scales are clearly visible. Include numbers along axes only for bottom left plot of group (a 'group' is an analysis of identical markers).
- [x] All plots are contour plots with outliers or pseudocolor plots.
- [x] A numerical value for number of cells or percentage (with statistics) is provided.

Methodology

Sample preparation	To assess neutrophil recruitment to the lung, murine lungss were removed, minced and digested for tissue dissociation. The suspension was washed in PBS and filtered through 70µm cell strainers Cells were stained with the specific antibodies and analyzed by Flow cytometry. To analyze cell viability isolated endothelial cells and neutrophils were washed with cold BioLegend's Cell Staining Buffer, and then resuspended in Annexin V Binding Buffer at a concentration of 0.25-1.0 x 107 cells/mL. Resuspended cells were stained and analyzed by Flow cytometry.
Instrument	BD FACSCanto™ II Flow Cytometry System
Software	To collect flow cytometry data the BD FACSDivaTM Software was used. For flow cytometry analyses we used the FlowJo™

	Software.
Cell population abundance	No post-sort fractions were collected.
Gating strategy	Negative control (unstained) and isotype controls were used to establish gates for each cell type. Gates were drawn to collect cells expressing either fluorophore. The manuscript contains all gates used.

☒ Tick this box to confirm that a figure exemplifying the gating strategy is provided in the Supplementary Information.
