# Supplementary material for: Alveolar epithelial and vascular CXCR2 mediates transcytosis of CXCL1 in inflamed lungs
Source: Nat Commun. 2025 May 24;16:4846. doi: 10.1038/s41467-025-60174-w (PMC12103508; doi:10.1038/s41467-025-60174-w)
Supplement: Supplementary file 3 — Lasing Reporting Summary [file 41467_2025_60174_MOESM3_ESM.pdf]

nature research

Corresponding author(s): DBPR NCOMMS-24-09401A

Lasing Reporting Summary

Nature Research wishes to improve the reproducibility of the work that we publish. This form is intended for publication with all accepted papers reporting claims of lasing and provides structure for consistency and transparency in reporting. Some list items might not apply to an individual manuscript, but all fields must be completed for clarity.

For further information on Nature Research policies, including our data availability policy, see Authors & Referees.

• Experimental design

Please check: are the following details reported in the manuscript?

1. Threshold

Plots of device output power versus pump power over a wide range of values indicating a clear threshold

- [ ] Yes
- [x] No

Study does not involve laser/lasing.

2. Linewidth narrowing

Plots of spectral power density for the emission at pump powers below, around, and above the lasing threshold, indicating a clear linewidth narrowing at threshold

- [ ] Yes
- [x] No

Study does not involve laser/lasing.

Resolution of the spectrometer used to make spectral measurements

- [ ] Yes
- [x] No

Study does not involve laser/lasing.

3. Coherent emission

Measurements of the coherence and/or polarization of the emission

- [ ] Yes
- [x] No

Study does not involve laser/lasing.

4. Beam spatial profile

Image and/or measurement of the spatial shape and profile of the emission, showing a well-defined beam above threshold

- [ ] Yes
- [x] No

Study does not involve laser/lasing.

5. Operating conditions

Description of the laser and pumping conditions
Continuous-wave, pulsed, temperature of operation

- [ ] Yes
- [x] No

Study does not involve laser/lasing.

Threshold values provided as density values (e.g. W cm^{-2} or J cm^{-2}) taking into account the area of the device

- [ ] Yes
- [x] No

Study does not involve laser/lasing.

6. Alternative explanations

Reasoning as to why alternative explanations have been ruled out as responsible for the emission characteristics
e.g. amplified spontaneous, directional scattering; modification of fluorescence spectrum by the cavity

- [ ] Yes
- [x] No

Study does not involve laser/lasing.

7. Theoretical analysis

Theoretical analysis that ensures that the experimental values measured are realistic and reasonable
e.g. laser threshold, linewidth, cavity gain-loss, efficiency

- [ ] Yes
- [x] No

Study does not involve laser/lasing.

8. Statistics

Number of devices fabricated and tested

- [ ] Yes
- [x] No

Study does not involve laser/lasing.

Statistical analysis of the device performance and lifetime (time to failure)

- [ ] Yes
- [x] No

Study does not involve laser/lasing.
